# Potential mechanisms of *Bifidobacterium*-mediated synergistic antibiotic eradication of drug-resistant *Helicobacter pylori*

**DOI:** 10.3389/fmicb.2026.1911939

**Published:** 2026-09-28

**Authors:** Linna Wu, Xin Chen, Mingyu Cui, Shuang Yan, Lijuan Wei, Ruifeng Duan

**Affiliations:** Department of Gastroenterology and Digestive Endoscopy Center, The Second Hospital of Jilin University, Changchun, China

**Keywords:** antibiotic resistance, bifidobacteria, *Helicobacter pylori*, probiotic, synbiotics

## Abstract

The rising prevalence of antibiotic-resistant *Helicobacter pylori* has become a major obstacle to the success of conventional eradication therapies, including triple and quadruple regimens combining antibiotics with proton pump inhibitors. There is an urgent need for safe and effective ad-junctive strategies to restore antibiotic efficacy. Probiotics, particularly *Bifidobacterium* species, have gained increasing attention for their potential to enhance *H. pylori* eradication and mitigate treatment-associated adverse effects. This review provides a comprehensive synthesis of the key molecular mechanisms driving *H. pylori* antibiotic resistance, including chromosomal mutations, altered expression of outer membrane proteins, enhanced efflux pump activity, biofilm formation, and morphological transition into dormant coccoid forms. Importantly, we critically examine the emerging evidence on how *Bifidobacterium* may synergize with antibiotics to counteract these resistance mechanisms. The proposed mechanisms include the secretion of bioactive metabolites (e.g., organic acids and antimicrobial peptides), reinforcement of the gastric mucosal barrier, modulation of host immune responses, and competitive exclusion of *H. pylori*. By integrating these mechanistic in-sights, this review highlights the potential of *Bifidobacterium* as a promising adjunctive therapeutic agent for overcoming antibiotic-resistant *H. pylori* infections and calls for more rigorous, strain-specific clinical investigations to translate these findings into practice.

## 1 Introduction

*Helicobacter pylori* (*H. pylori*) is a Gram-negative, microaerophilic pathogen that colonizes the human gastric mucosa and is etiologically linked to a spectrum of gastrointestinal disorders, including chronic gastritis, peptic ulcer disease, and gastric adenocarcinoma (Patel et al., 2026). Beyond the stomach, *H. pylori* infection has also been associated with various extra gastric conditions, such as non-alcoholic fatty liver disease and osteoporosis (Zhang et al., 2024; Xiong et al., 2023; Liu et al., 2023). *H. pylori* infection remains common worldwide, although its prevalence varies greatly between regions. A recent global meta-analysis showed that the prevalence of *H. pylori* infection was about 43.9% in adults and 35.1% in children and adolescents during 2015–2022 (Chen et al., 2024). The prevalence is generally higher in low- and middle-income regions, where sanitation, living conditions, and access to medical care may be limited. This large infected population makes effective *H. pylori* eradication an important clinical issue.

Current guidelines recommend combination regimens that include acid suppression with one or more antibiotics, with or without bismuth, for *H. pylori* eradication. The choice of treatment depends on local antibiotic resistance patterns, previous antibiotic exposure, and antimicrobial susceptibility testing when available. Susceptibility-guided therapy is increasingly recommended, especially when clarithromycin- or fluoroquinolone-containing regimens are considered (Malfertheiner et al., 2022; Chey et al., 2024). However, antibiotic resistance has reduced the effectiveness of many traditional regimens. Resistance to clarithromycin, metronidazole, and levofloxacin is now common in many countries. The problem is especially important because these drugs have been widely used in *H. pylori* treatment for many years.

The prevalence of antibiotic resistance differs between regions, but high resistance rates have been reported worldwide. In the Asia-Pacific region, a meta-analysis covering data from 1990 to 2022 showed that recent primary resistance rates were about 30% for clarithromycin, 61% for metronidazole, and 35% for levofloxacin (Chey et al., 2024). European registry data also showed high resistance rates in treatment-naïve patients. The reported rates were about 25% for clarithromycin, 30% for metronidazole, and 20% for levofloxacin (Bujanda et al., 2021). In Africa, a recent meta-analysis estimated that clarithromycin resistance was about 27% (Dossouvi et al., 2025). In the United States, current clinical guidelines report clarithromycin resistance rates of about 20–30%, while levofloxacin resistance may approach 40% (Chey et al., 2024). These results show that resistance is not limited to one country or one population. Resistance has become a common problem in both developed and developing regions.

Antibiotic resistance has a direct effect on treatment success. Clarithromycin resistance is a clear example. A large systematic review that included more than 66,000 *H. pylori* isolates found that clarithromycin resistance was associated with a significantly increased risk of treatment failure. Clarithromycin resistance markedly reduces the efficacy of clarithromycin-containing eradication regimens (Chey et al., 2024; Bujanda et al., 2021). In patients infected with clarithromycin-resistant strains, the eradication rate of conventional clarithromycin-based triple therapy may fall to about 30% (Chey et al., 2024). Treatment failure can also make later treatment more difficult. European registry data showed that more than 80% of previously treated patients had resistance to at least one antibiotic. After failure of first-line treatment, clarithromycin resistance exceeded 60%. Dual resistance to clarithromycin and metronidazole was found in about 43% of patients, while triple resistance to clarithromycin, metronidazole, and levofloxacin was found in about 19% (Bujanda et al., 2021). Repeated antibiotic exposure may further increase the chance of selecting resistant strains. Patients may then require more complex rescue regimens. These regimens often include more drugs or longer treatment courses. They may also increase adverse effects such as diarrhea, nausea, abdominal discomfort, and changes in the intestinal microbiota.

However, the widespread and often inappropriate use of antibiotics has fueled a relentless rise in *H. pylori* resistance, directly undermining treatment success. This resistance is accompanied by increased rates of adverse effects, including antibiotic-associated diarrhea, nausea, and other gastrointestinal symptoms (Bordin et al., 2022; Asgari et al., 2020; Tanashat et al., 2025). Consequently, antibiotic resistance has emerged as a major factor limiting successful eradication and complicating *H. pylori* management, thereby spurring the search for novel therapeutic strategies. These include potassium-competitive acid blockers (e.g., vonoprazan) and host-derived antimicrobial peptides, which have shown promise against drug-resistant strains (Chey et al., 2022; Ouyang et al., 2023; Roszczenko-Jasińska et al., 2020; Kiyotoki et al., 2020; Shinozaki et al., 2021; Du R. C. et al., 2024).

Probiotics have also received increasing attention because they are generally well tolerated and may support conventional eradication therapy. Probiotics are live microorganisms that can provide health benefits when they are given in adequate amounts (Gupta et al., 2023; Yuan et al., 2025). They can be given as capsules, powders, fermented foods, or other preparations. Several probiotic groups have been studied in *H. pylori* infection. These include *Lactobacillus*, *Bifidobacterium*, and some yeast strains (Yuan et al., 2025). Accumulating evidence suggests that *Bifidobacterium* supplementation may improve outcomes for patients with antibiotic-resistant *H. pylori* infection (Yuan et al., 2025; He et al., 2022; Viazis et al., 2022). However, evidence in patients infected with antibiotic-resistant *H. pylori* is still limited. The mechanisms by which *Bifidobacterium* may increase the antibacterial effect of antibiotics also remain unclear (Yuan et al., 2025). This review focuses on the possible mechanisms through which *Bifidobacterium* may enhance antibiotic eradication of drug-resistant *H. pylori*, with particular attention to direct antibacterial effects, inhibition of bacterial colonization, regulation of host immune responses, protection of the gastric mucosal barrier, and changes in the gastrointestinal microbial environment.

## 2 Mechanisms of antibiotic resistance in *Helicobacter pylori*

To reduce overlap among individual resistance mechanisms, the major determinants of *H. pylori* antibiotic resistance can be organized into five interacting modules: (1) antibiotic target modification and impaired drug activation; (2) reduced drug influx and enhanced drug efflux; (3) biofilm-associated tolerance and quorum-sensing regulation; (4) morphological transition to the coccoid or viable but non-culturable state; and (5) virulence-associated bacterial phenotypes that may modify treatment response. These mechanisms are not completely independent and may interact during persistent infection and antibiotic exposure (Lin Y. et al., 2023; Tshibangu-Kabamba and Yamaoka, 2021; Krzyżek et al., 2020; Fauzia et al., 2024; Srisuphanunt et al., 2023; Medakina et al., 2023; Ansari and Yamaoka, 2022; Liu et al., 2022; Krzyżek, 2024; Zhou et al., 2026; Sadeghloo et al., 2025; El Mortaji et al., 2020; Ierardi et al., 2020; Kadkhodaei et al., 2020) (Table 1; Figure 1).

**Table 1:** Major molecular and phenotypic mechanisms associated with antibiotic resistance and tolerance in *H. pylori*.

Resistance category	Molecular determinant(s)	Mechanism of resistance	Antibiotic(s) affected	References
Target modification and impaired drug activation	23S rRNA	Point mutations diminish ribosomal affinity for clarithromycin	Clarithromycin	Lin Y. et al. (2023), Srisuphanunt et al. (2023), Medakina et al. (2023), Ansari and Yamaoka (2022), Puah et al. (2021), and Ziver-Sarp et al. (2021)
	*gyrA, gyrB*	Altered DNA gyrase and reduced fluoroquinolone susceptibility	Levofloxacin	Lin Y. et al. (2023), Medakina et al. (2023), and Ansari and Yamaoka (2022)
	*rpoB*	Altered RNA polymerase β-subunit	Rifamycins	Srisuphanunt et al. (2023), Medakina et al. (2023), and Ng et al. (2023)
	*porD/oorD*	Reductase-associated alterations implicated in resistance	Furazolidone	Srisuphanunt et al. (2023) and Medakina et al. (2023)
	*rdxA/frxA*	Reduced activation of metronidazole	Metronidazole	Lin Y. et al. (2023), Srisuphanunt et al., 2023; Medakina et al. (2023), and Ansari and Yamaoka (2022)
	*pbp1A*	Altered penicillin-binding protein and reduced amoxicillin affinity	Amoxicillin	Tran et al. (2022), Tseng et al. (2009), and Qureshi et al. (2014)
Reduced drug influx and enhanced drug efflux	HopD, HopE and other porins	Reduced porin-mediated drug influx and decreased intracellular antibiotic accumulation	Multiple antibiotics	Liu et al. (2022)
	HefA and other efflux systems	Reduced intracellular antibiotic concentration	Multidrug resistance	Krzyżek (2024) and Gong et al. (2024)
Biofilm-associated tolerance and quorum sensing	EPS, extracellular DNA, and proteins	Restricted antibiotic penetration and reduced metabolic activity	Multidrug tolerance	Qureshi et al. (2014) and Guo et al. (2020)
	*luxS*/AI-2	Regulation of chemotaxis, spatial organization, and biofilm behavior; direct role in resistance remains incompletely defined	Indirect/phenotypic tolerance	Fauzia et al. (2024) and Krzyżek (2024)
Coccoid/VBNC transition	Coccoid/VBNC phenotype	Reduced metabolic activity, persistence under stress, potential reactivation	Reduced antibiotic susceptibility/persistence	Sadeghloo et al. (2025) and Ramezani et al. (2025)
Virulence-associated phenotypes	CagA, VacA	Associated with resistance patterns in some populations; not established as direct resistance determinants	Epidemiological association	Karbalaei et al. (2022)

Chromosomal target mutations represent established antibiotic-resistance mechanisms, whereas biofilm formation, quorum sensing, coccoid/VBNC transition, and virulence-associated phenotypes may contribute to antibiotic tolerance, persistence, or treatment failure. The direct causal role of CagA, VacA, and AI-2 signaling in antimicrobial resistance remains incompletely defined. EPS: Extracellular polymeric substance.

**Figure 1:**
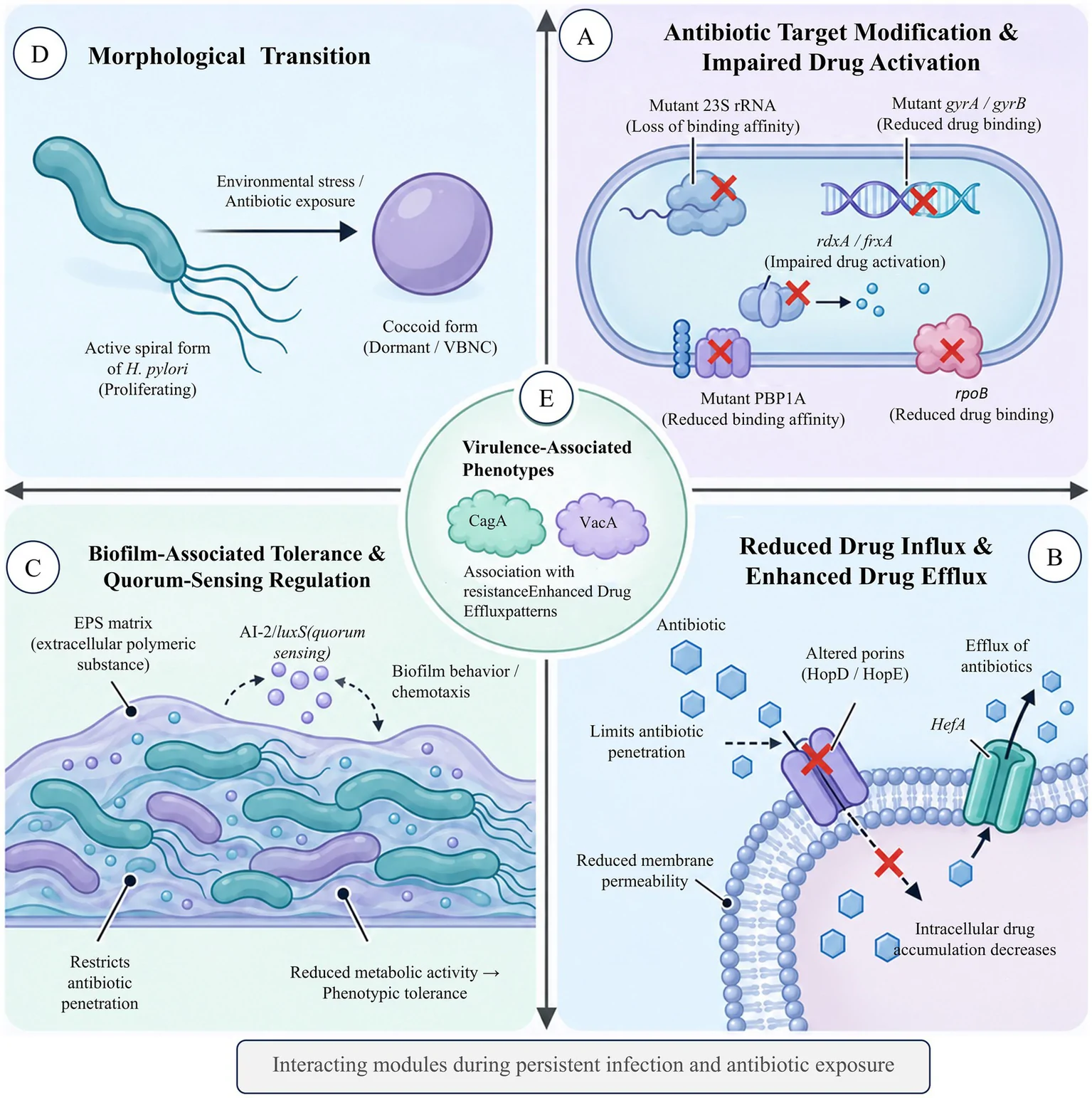
Major molecular and phenotypic mechanisms associated with antibiotic resistance and tolerance in *H. pylori*. **(A)** Morphological transition: under environmental stress or antibiotic exposure, actively proliferating spiral-form *H. pylori* can convert to coccoid or viable but non-culturable (VBNC) forms with markedly reduced metabolic activity. **(B)** Antibiotic target modification and impaired drug activation: mutations in 23S rRNA, *gyrA/gyrB, rpoB, rdxA/frxA*, and *pbp1A* can reduce antibiotic binding or impair drug activation. **(C)** Biofilm-associated tolerance and quorum-sensing regulation: the EPS matrix restricts antibiotic penetration and supports phenotypically tolerant bacterial populations, while *luxS*-dependent AI-2 signaling contributes to chemotaxis and biofilm regulation. **(D)** Reduced drug influx and enhanced drug efflux: altered outer-membrane porins, including HopD and HopE, can decrease antibiotic entry, whereas efflux systems such as HefA reduce intracellular drug accumulation. (5) Virulence-associated phenotypes: CagA and VacA have been associated with antibiotic resistance patterns in some populations but are not established as direct resistance determinants.

### 2.1 Antibiotic target modification and impaired drug activation

Chromosomal mutations remain the best-established molecular basis of *H. pylori* antibiotic resistance (Srisuphanunt et al., 2023; Schulz et al., 2025). Mutations in domain V of 23S rRNA reduce clarithromycin binding to the 50S ribosomal subunit, whereas mutations in *gyrA* and, less commonly, *gyrB* alter DNA gyrase and reduce fluoroquinolone susceptibility (Lin Y. et al., 2023; Srisuphanunt et al., 2023; Medakina et al., 2023; Ansari and Yamaoka, 2022). Mutations in *rpoB* are associated with rifamycin resistance, while alterations in reductase-associated genes have been implicated in resistance to nitroimidazoles and furazolidone (Srisuphanunt et al., 2023). Loss-of-function mutations in *rdxA* and *frxA* reduce activation of metronidazole (Lin Y. et al., 2023; Resina and Gisbert, 2021). Amoxicillin resistance is mainly associated with alterations in *pbp1A* and other determinants affecting cell-wall synthesis and drug access (Lin Y. et al., 2023; Tran et al., 2022).

### 2.2 Reduced drug influx and enhanced drug efflux

Changes in outer membrane proteins and membrane permeability may reduce antibiotic entry into *H. pylori*. In particular, altered function or loss of porins such as HopD and HopE can decrease intracellular antibiotic accumulation. Efflux systems provide a complementary mechanism by actively reducing intracellular drug concentrations. Recent evidence also suggests interactions between efflux activity, resistance-associated mutations, and biofilm formation (Liu et al., 2022; Krzyżek, 2024; Zhou et al., 2026). These mechanisms are therefore considered together rather than as independent resistance pathways.

Efflux pumps reduce intracellular antibiotic concentrations and may contribute to both antimicrobial resistance and biofilm-associated tolerance. Recent studies also suggest interactions between efflux activity and resistance-related mutations (Krzyżek, 2024; Gong et al., 2024).

### 2.3 Biofilm-associated tolerance and quorum-sensing regulation

Biofilm formation provides *H. pylori* with a protected ecological state rather than a single antibiotic-specific resistance mechanism. The extracellular polymeric matrix can restrict antibiotic penetration, while reduced bacterial metabolic activity within mature biofilms may decrease susceptibility to antibiotics that preferentially act on actively growing cells. Biofilms may also facilitate persistent bacterial populations and are closely associated with efflux activity (Fauzia et al., 2024; Krzyżek, 2024).

Quorum sensing contributes to the regulation of *H. pylori* population behavior and biofilm dynamics. The *luxS*-dependent signaling molecule AI-2 participates in chemotaxis, spatial organization, and biofilm regulation (Lin Y. et al., 2023). However, current evidence does not establish a simple causal pathway in which AI-2 directly activates efflux pumps or induces the coccoid transition. The relationships among AI-2 signaling, biofilm maturation, efflux activity, and morphological adaptation should therefore be viewed as interconnected but incompletely defined processes rather than as a single linear resistance pathway (Fauzia et al., 2024; Krzyżek, 2024).

### 2.4 Coccoid and viable but non-culturable states

Under environmental stress or antibiotic exposure, *H. pylori* can transition from the metabolically active spiral form to a coccoid or viable but non-culturable state (Sadeghloo et al., 2025; El Mortaji et al., 2020). These cells show markedly reduced metabolic activity and may exhibit increased tolerance to antimicrobial treatment (Ierardi et al., 2020; Kadkhodaei et al., 2020). Coccoid forms can persist under unfavorable conditions and may regain biological activity when environmental conditions improve (Ramezani et al., 2025; Li et al., 2024). Whether probiotics or their metabolites can prevent the spiral-to-coccoid transition, directly eliminate established coccoid cells, or prevent their reactivation has not yet been established.

### 2.5 Virulence factors CagA and VacA as resistance-associated phenotypes

CagA and VacA are major *H. pylori* virulence factors rather than canonical antibiotic resistance determinants. Nevertheless, several studies have reported associations between virulence genotypes and antimicrobial susceptibility (Zhao et al., 2023; Shetty et al., 2021). A recent systematic review and meta-analysis found that *cagA*-positive strains were associated with increased metronidazole resistance in some populations, while the *vacA* s1m1 genotype was associated with resistance to several antibiotics in selected geographic subgroups (Karbalaei et al., 2022). However, these associations were heterogeneous and do not establish a direct molecular role of CagA or VacA in antibiotic resistance. Virulence-associated inflammation, epithelial injury, bacterial fitness, strain genetic background, and biofilm behavior may partly explain these observations. Further studies are required to distinguish causal mechanisms from epidemiological linkage.

Table 1 summarizes the major genetic and phenotypic mechanisms that contribute to antibiotic resistance in *H. pylori*. These mechanisms include mutations in antibiotic target genes or drug-activating enzymes, changes in outer membrane proteins, increased drug efflux, biofilm formation, and other factors that may contribute to reduced antimicrobial susceptibility. The table also lists the main molecular determinants associated with each mechanism and the antibiotics that may be affected. Some resistance mechanisms are antibiotic-specific, whereas others, such as increased efflux and biofilm formation, may contribute to multidrug resistance. The contribution of individual determinants may vary among *H. pylori* strains, and some proposed mechanisms require further clinical validation.

## 3 *Helicobacter pylori* and gut microbiota

Gut Microbiota Dysbiosis and Resistance: *H. pylori* infection perturbs the equilibrium of gut microbiota, potentially affecting antibiotic resistance through modifications in host immunity and defense mechanisms (Srisuphanunt et al., 2023; Gong et al., 2021). While extensive knowledge exists on *H. pylori* resistance mechanisms, certain molecular details are still elusive (Zhang et al., 2010). Ongoing research is imperative to elucidate these mechanisms and formulate effective strategies to confront the escalating problem of *H. pylori* resistance.

The human gut harbors a highly dynamic microbial ecosystem that maintains mucosal homeostasis, reinforces epithelial integrity, and orchestrates local immunity—a collective defense known as colonization resistance. Disruption of this ecosystem, or dysbiosis, particularly depletion of short-chain fatty acid (SCFA)-producing commensals such as *Bifidobacterium* and *Lactobacillus*—is increasingly recognized as a key predisposing factor for *H. pylori* colonization (Li et al., 2024; Nabavi-Rad et al., 2022).

Mechanistically, SCFA deficiency compromises host defense on three levels. First, the loss of direct microbial antagonism removes organic acid-mediated inhibition of *H. pylori* urease activity, an enzyme essential for bacterial survival in the acidic gastric lumen (Malfertheiner et al., 2023). Second, SCFAs can support epithelial barrier function, and their reduction may contribute to mucus barrier disruption and increased epithelial permeability, facilitating *H. pylori* adhesion and penetration (Tanashat et al., 2025; Yuan et al., 2025). Third, SCFA scarcity impairs regulatory T cell differentiation and skews the local immune balance toward a pro-inflammatory Th1/Th17 phenotype. Paradoxically, this chronic inflammation creates an opportunistic niche that supports persistent *H. pylori* colonization (Xu W. et al., 2022).

Importantly, *Bifidobacterium* can counteract these dysbiosis-driven vulnerabilities. By producing organic acids and immunomodulatory metabolites, *Bifidobacterium* attenuates *H. pylori*-induced NF-κB and MAPK signaling, reducing pro-inflammatory cytokines (IL-1, IL-6, IL-8, TNF-α) and limiting gastric mucosal injury (Yuan et al., 2025; Zheng et al., 2024; Zhang et al., 2023; Guo et al., 2026; Yu et al., 2024). Additionally, *Bifidobacterium* may restore mucosal integrity by upregulating tight junction proteins and mucin secretion—mechanisms well established in the lower gut and plausibly applicable to the *H. pylori*-damaged gastric epithelium (Li et al., 2019; Xu B. et al., 2022; Pang et al., 2022; Yue et al., 2023; Jung et al., 2022). In parallel, other commensals such as *Lactobacillus* may modulate inflammatory signaling, further suppressing *H. pylori*-driven inflammation (Yuan et al., 2025). Thus, restoring keystone commensals represents a rational adjunctive strategy to re-establish mucosal homeostasis before or during antibiotic therapy.

## 4 Current and emerging therapeutic strategies for *Helicobacter pylori* infection

Current treatment of *H. pylori* infection still relies mainly on antibiotic-based combination regimens, but their efficacy is increasingly compromised by resistance, particularly to clarithromycin, metronidazole, and levofloxacin (Chey et al., 2024; Krzyżek, 2024; Hong et al., 2024; Huang et al., 2024; Lee et al., 2022). Previous antibiotic exposure and local resistance patterns should also be considered when a regimen is selected (Malfertheiner et al., 2022; Chey et al., 2024). Molecular resistance testing provides another way to guide antibiotic selection. A recent meta-analysis showed that genotypic susceptibility-guided therapy achieved pooled eradication rates of 86.9% in intention-to-treat analysis and 91.5% in per-protocol analysis (Lin K. et al., 2023). This approach may reduce the use of antibiotics that are unlikely to be effective.

However, these regimens still depend mainly on antibiotics, and increasing resistance continues to limit treatment success. For this reason, several new approaches are being studied to reduce dependence on conventional antibiotics. Potassium-competitive acid blockers, especially vonoprazan, provide stronger and more stable acid suppression than conventional PPIs. This property has supported the development of vonoprazan-based dual and triple regimens (Huang et al., 2024). Other strategies target *H. pylori* through mechanisms that differ from those of conventional antibiotics. Antimicrobial peptides can damage bacterial membranes and may retain activity against some resistant strains. Nanoparticles and other targeted delivery systems can increase local drug concentrations in the gastric mucus and may reduce systemic drug exposure (Chitas et al., 2024). Photodynamic therapy can kill *H. pylori* through the production of reactive oxygen species, while bacteriophages and phage-derived products can target bacterial cells through specific biological mechanisms (Luo et al., 2023; Luo et al., 2024). Vaccines, postbiotics, and other biomaterial-based approaches are also under investigation (Chitas et al., 2024; Yunle et al., 2024; Ivashkin et al., 2024). These approaches may bypass some common mechanisms of antibiotic resistance. However, most of them are still supported mainly by laboratory studies, animal studies, or small clinical studies. More clinical evidence is needed before they can be used routinely. At present, adjunctive strategies that can be combined with established eradication regimens may therefore provide a more practical way to improve treatment outcomes.

In this context, probiotics have emerged as a promising adjunctive strategy. Supplementation with probiotics, especially *Bifidobacterium* species, has been shown to improve eradication rates and alleviate antibiotic-induced gastrointestinal side effects, such as diarrhea and nausea (Malfertheiner et al., 2023; Fauzia and Tuan, 2024; Yang et al., 2024). Among bifidobacteria, different strains exhibit distinct properties: *B. longum* enhances digestive and immune health while antagonizing *H. pylori* (Dong et al., 2022). *B. infantis* exerts anti-inflammatory effects (Wang I. K. et al., 2021; Chen et al., 2023), and *B. bifidum* competes for binding sites and has shown particular efficacy in *H. pylori* eradication (Lin et al., 2022) (Table 2). These strain-specific effects provide the basis for considering *Bifidobacterium* as an adjunct to antibiotic therapy, especially when conventional treatment is limited by resistance or treatment-related adverse effects.

**Table 2:** Main characteristics and potential therapeutic roles of selected *Bifidobacterium* species in *H. pylori* management.

Species/Preparation	Evidence relevant to *H. pylori*	References
*Bifidobacterium longum*	Inhibits *H. pylori* growth and may attenuate gastric inflammation in experimental models	Shin et al. (2024)
*B. animalis subsp. lactis* BB18	Reduced *H. pylori* burden, gastric mucosal injury, and inflammatory cytokines in experimental models	Zheng et al. (2024)
*Bifidobacterium*-containing probiotic preparations	May improve eradication outcomes and reduce treatment-related adverse events as adjuncts to standard therapy.	Tanashat et al. (2025), Ivashkin et al. (2024), and Yang et al. (2024)

## 5 Therapeutic role of *Bifidobacterium* in *Helicobacter pylori* infection

In recent years, the role of probiotics as adjunctive therapy for *H. pylori* infection has been increasingly recognized (Table 3). Animal studies have shown that both *Bifidobacterium longum* P142 and *Lactococcus lactis* P135 can inhibit *H. pylori* growth and attenuate *H. pylori*-induced gastric inflammation (Shin et al., 2024). Moreover, *Bifidobacterium longum* has been noted to alleviate *H. pylori*-induced intestinal ecological dysregulation, underscoring its potential as adjunctive therapy (Du L. et al., 2024).

**Table 3:** Recent clinical studies evaluating *Bifidobacterium*-containing probiotics as adjuncts to *H. pylori* eradication therapy.

Author & year	Study design, sample size and country	Probiotic formulation and timing	Background eradication regimen	Eradication assessment and result	Other outcomes
Jiang et al. (2025)	Single-center, randomized, single-blind trial; adults with *H. pylori*-positive gastric ulcer; mean age approximately 46 years;	Bifidobacteria Quadruple Viable Bacteria Tablets for 2 weeks, concomitant with eradication therapy	Esomeprazole + Colloidal bismuth pectin + Clarithromycin + Amoxicillin	^14C-UBT after treatment; 92.0% vs. 78.0%; analysis set not explicitly labeled ITT/PP	Lower adverse-event rate and inflammatory markers in probiotic group
Han et al. (2024)	Single-center, randomized, double-blind, placebo-controlled trial; treatment-naïve adults aged 18–70 years;	*B. animalis* subsp. *lactis* BLa80, 1 × 10^11 CFU/day for 14 days, concomitant with therapy and separated from antibiotics by ≥2 h	Esomeprazole + Bismuth + Amoxicillin + Clarithromycin	ITT: 86.0% vs. 86.0%; PP: 87.76% vs. 86.0%; no significant difference	Less nausea and greater improvement in GSRS
Wang et al. (2024)	Single-center, open-label, randomized four-arm trial; treatment-naïve adults;	Synbiotic containing three *Bifidobacterium* strains, three *Lactobacillus* strains and dietary fiber for 6 weeks in relevant arms	Rabeprazole + Minocycline +Amoxicillin+ Bismuth	^13C-UBT at week 6; QT/QF/QS/QFS: 95%/100%/90%/95%; no significant difference	Synbiotic/fucoidan-containing regimens modified gut microbiota responses
Peng et al. (2023)	Single-center, randomized, double-blind, placebo-controlled trial; adults aged 18–65 years;	Tetragenous Viable Bacteria Tablets, 3 tablets three times daily for 14 days, separated from amoxicillin by >2 h	Vonoprazan + Amoxicillin	Study focused on oral microbiota rather than comparative eradication efficacy; only successfully eradicated patients entered longitudinal microbiota analysis	Probiotics did not prevent eradication-associated oral microbiota disturbance
He et al. (2022)	Multicenter, randomized, double-blind, placebo-controlled trial; treatment-naïve patients;	Bifidobacterium Tetragenous Viable Bacteria Tablets for 28 days; antibiotics given for 14 days	Esomeprazole + Bismuth + Amoxicillin + Furazolidone	No significant difference in eradication rate	GI adverse events: 23.6% vs. 37.7%; reduced microbiota disturbance
Viazis et al. (2022)	Multicenter, prospective, randomized, placebo-controlled trial; mean age 51.6 vs. 49.9 years;	Four-strain probiotic containing *L. acidophilus*, *Lactiplantibacillus plantarum*, *B. lactis*, and *S. boulardii*, administered with therapy	Omeprazole + Amoxicillin + Clarithromycin + Metronidazole	PP: 92.0% vs. 86.8%, *p* = 0.028; ITT not used because outcomes were unavailable for patients lost to follow-up	Treatment-associated symptoms: 17.0% vs. 50.7%
Yuan et al. (2021)	Randomized intervention study in young adults aged 19–30 years;	*Bifidobacterium*-containing probiotic intervention	Omeprazole + Amoxicillin + Clarithromycin + Bismuth	Study primarily evaluated gastric microbiota rather than eradication efficacy	Probiotics partially restored gastric dysbiosis; monotherapy showed no clear advantage in *H. pylori* inhibition
Yakovenko et al. (2021)	Open, prospective, randomized comparative trial; peptic ulcer/chronic gastritis/gastroduodenitis;	*B. longum* BB-46 + *E. faecium* ENCfa-68 for 14 days	Omeprazole + Amoxicillin + Clarithromycin + Bismuth	>90% eradication in PP analysis in probiotic group	Fewer adverse effects, lower dropout rate, and improvement in colonic dysbiosis

CFU, colony-forming units; GI, gastrointestinal; GSRS, Gastrointestinal Symptom Rating Scale; ITT, intention-to-treat; NR, not reported; PP, per-protocol; RCT, randomized controlled trial; UBT, urea breath test.

Recent clinical evidence supports the use of *Bifidobacterium*-containing preparations mainly as adjuncts rather than stand-alone eradication treatments. A 2023 systematic review and meta-analysis found that *Bifidobacterium*-containing viable bacterial tablets combined with bismuth quadruple therapy were associated with higher eradication rates and fewer adverse reactions than quadruple therapy alone (Jiang et al., 2023). Recent clinical trials have also evaluated different Bifidobacterium-containing preparations, although their effects on eradication rates have not been consistent across studies (He et al., 2022; Viazis et al., 2022; Yang et al., 2024; Jiang et al., 2025; Wang et al., 2024; Peng et al., 2023; Yuan et al., 2021).

Despite these promising therapeutic roles, several clinical issues require further investigation. The identification of the most effective *Bifidobacterium* strains and optimization of the timing, dose, and duration of administration remain important areas for future research (Tanashat et al., 2025; Yuan et al., 2025). In addition, postbiotics may provide an alternative strategy that avoids some of the viability and stability limitations associated with live probiotics (Yan et al., 2024). Postbiotics, including non-viable microbial preparations and biologically active microbial products, do not require persistent bacterial survival during antibiotic exposure or in the acidic gastric environment. Their potential effects may be mediated through microbial metabolites, antimicrobial compounds, cell-surface components, or immunomodulatory molecules. However, direct evidence demonstrating that *Bifidobacterium*-derived postbiotics enhance eradication of antibiotic-resistant *H. pylori* remains limited, and this possibility requires further experimental and clinical validation.

### 5.1 Promotion of organic acid production and potential antibacterial effects

*Bifidobacterium* spp. can produce organic acids and other metabolites that contribute to inhibition of potentially harmful bacteria (Yuan et al., 2025). Similar to *Lactobacillus*, *Bifidobacterium* produces lactic acid, which may contribute to an unfavorable microenvironment for *H. pylori* (Goderska et al., *2018*). Recent studies have indicated that zinc acetate can increase the sensitivity of *H. pylori* to levofloxacin (Tao et al., 2022), providing a potential mechanism through which *Bifidobacterium* spp. might aid in *H. pylori* antibiotic treatment by promoting acetate production (Jiang et al., 2023). However, because many *Bifidobacterium* strains are primarily adapted to the intestinal environment rather than persistent high-abundance colonization of the stomach, whether these metabolites reach sufficient concentrations in the gastric niche to substantially alter intragastric pH or directly enhance antibiotic activity remains uncertain. Therefore, the contribution of bifidobacterial organic acids to antibiotic sensitization of *H. pylori* should currently be regarded as a potential mechanism requiring direct experimental confirmation (Figure 2).

**Figure 2:**
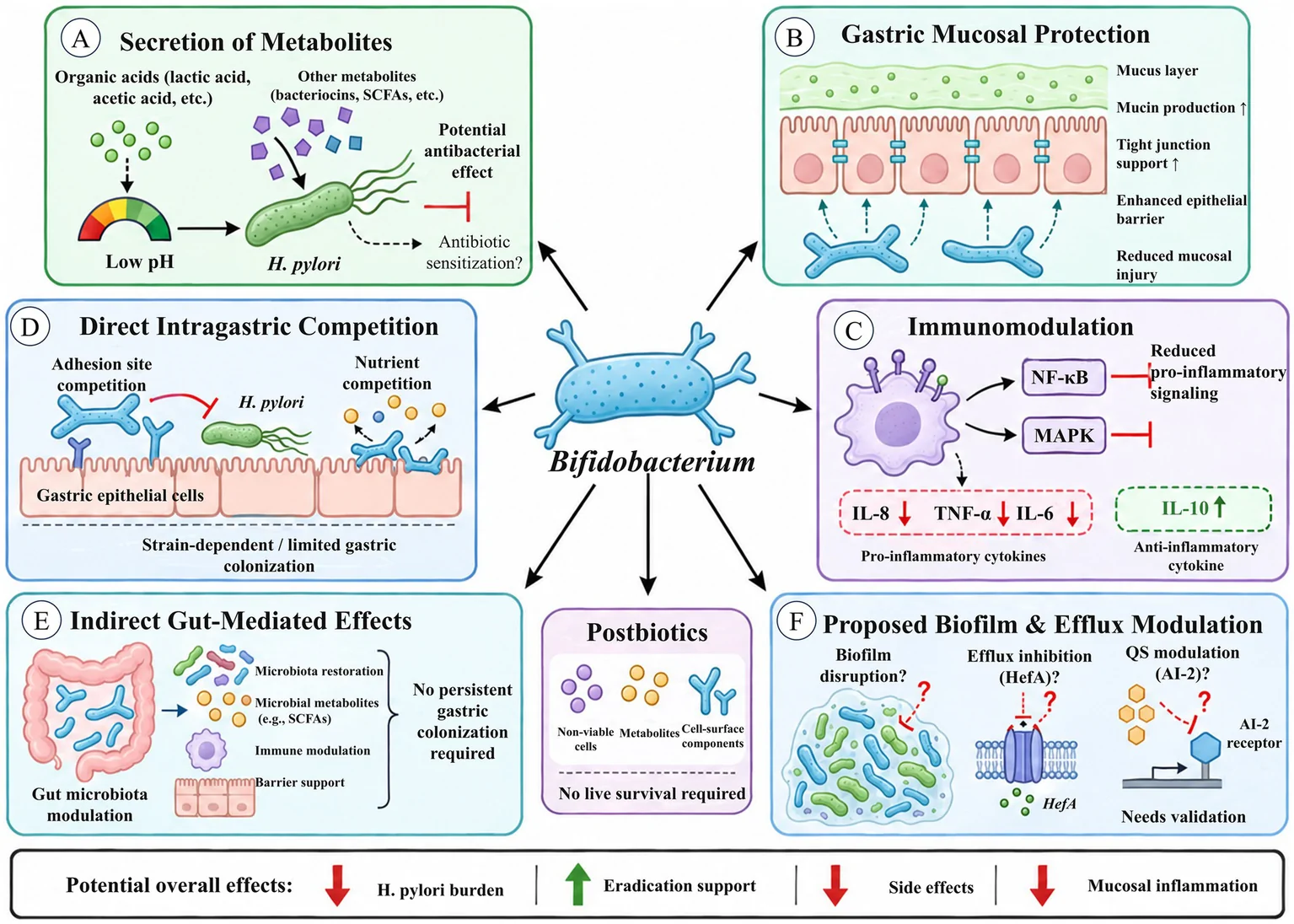
Mechanisms of *Bifidobacterium*-mediated enhancement of antibiotic efficacy against drug-resistant *H. pylori*. **(A)** Secretion of metabolites: organic acids and other microbial products may directly inhibit *H. pylori* growth, although their role in restoring antibiotic susceptibility remains incompletely defined. **(B)** Gastric mucosal protection: selected bifidobacterial strains may support mucus-associated barrier function, epithelial integrity, and protection against gastric mucosal injury. **(C)** Immunomodulation: *Bifidobacterium* may attenuate excessive NF-κB- and MAPK-related inflammatory signaling, reduce pro-inflammatory cytokines such as IL-8, TNF-α, and IL-6, and promote anti-inflammatory responses including IL-10. **(D)** Direct intragastric competition: selected strains may compete with *H. pylori* for adhesion sites or local nutrients; however, this mechanism is likely to be strain-dependent because persistent high-abundance gastric colonization by bifidobacteria may be limited. **(E)** Indirect gut-mediated effects: modulation of the distal gut microbiota, microbial metabolites, host immune responses, and epithelial barrier function may indirectly influence the broader gastrointestinal environment without requiring persistent gastric colonization. **(F)** Proposed biofilm and efflux modulation: interference with *H. pylori* biofilm formation, HefA-mediated efflux, or AI-2 quorum sensing remains biologically plausible but has not been directly established and therefore requires further molecular validation.

### 5.2 Production of antimicrobial peptides that directly inhibit *Helicobacter pylori* growth

Antimicrobial peptides and related microbial products have been proposed as potential approaches for suppressing *H. pylori*, including resistant strains (Tanashat et al., 2025). In addition to antimicrobial peptides, bifidobacteria can produce organic acids and other metabolites that may further suppress *H. pylori* growth and colonization (Gupta et al., 2023; Choudhury et al., 2023). However, direct evidence showing that naturally produced *Bifidobacterium*-derived antimicrobial peptides specifically restore clarithromycin or metronidazole susceptibility in resistant *H. pylori* remains limited.

### 5.3 Involvement in gastric mucosal protection and inhibition of *Helicobacter pylori* -induced inflammatory response

*Bifidobacterium* may contribute to gastric mucosal protection by interacting with the mucus layer, supporting epithelial barrier function, and reducing *H. pylori*-induced mucosal injury. Recent evidence suggests that bifidobacteria can promote mucus-associated barrier function, while selected strains have been shown to reduce gastric mucosal damage and inflammatory responses during *H. pylori* infection (Zheng et al., 2024; Guo et al., 2026; Gutierrez et al., 2023). B*ifidobacterium* may also modulate both innate and adaptive immune responses. Changes in inflammatory mediators such as IL-8, IL-10, IL-12, IL-23, and TNF-α have been reported in experimental studies of probiotic–*H. pylori* interactions (Yuan et al., 2025; Zhang et al., 2008). In addition, NF-κB and MAPK signaling represent important pathways involved in *H. pylori*-induced inflammation, and selected bifidobacterial strains may attenuate excessive activation of these inflammatory pathways (Figure 2) (Shirasawa et al., 2010; Yuan et al., 2025; Krzyżek, 2024; Xiang et al., 2025).

### 5.4 Formation of efflux pumps and inhibition of *Helicobacter pylori* biofilm formation

Efflux pumps constitute a critical bacterial resistance mechanism against antibiotics, effectively reducing intracellular drug concentration by expelling drugs. As classified by Fatahi-Bafghi et al. (Fatahi-Bafghi et al., 2022), bacterial efflux pumps are divided into five groups according to their structure, mechanism, and energy source: ATP-binding cassette (ABC) transporters, small multidrug resistance (SMR) pumps, resistance-nodulation-division (RND) family proteins, major facilitator superfamily (MFS) pumps, and MATE family pumps (Krzyżek, 2024). Particularly, MDR pumps are noted for their ability to recognize multiple drugs, and the genes encoding them are typically situated on mobile genetic elements such as plasmids or transposons, enabling transmission between strains through horizontal gene transfer. Probiotics bearing such genes could hypothetically convey resistance to commensal flora (e.g., *H. pylori*), representing a potential risk in clinical settings (Shahali et al., 2023; Rozman et al., 2023). However, not every drug-resistance gene entails a transfer risk. Because probiotics are administered during antibiotic therapy, their resistome also requires safety assessment. Genomic studies indicate that the risk of horizontal transfer depends on the identity of the resistance determinant and its association with mobile genetic elements (Ronkainen et al., 2025). This feature positions *Bifidobacterium bifidum* as an ideal adjunctive treatment for *H. pylori*: it obstructs *H. pylori* colonization through competitive nutrition without intensifying *H. pylori* resistance issues. Additionally, *H. pylori* resistance is intimately linked to its ability to form biofilms. *H. pylori* biofilms are composed of live/dead bacteria, polysaccharides, extracellular DNA, and proteins, establishing a dense structure that obstructs antibiotic penetration. The subdued metabolic activity of bacteria within biofilms further diminishes the drug’s efficacy (Fauzia et al., 2024). When efflux pump functionality is curtailed (e.g., reduced drug efflux), bacteria face challenges in forming stable biofilms due to the accumulation of intracellular toxic substances (Fatahi-Bafghi et al., 2022). It has been hypothesized that bifidobacteria or their metabolites may interfere with *H. pylori* biofilm formation through competition for nutrients or modulation of bacterial stress responses (Fauzia et al., 2024; Krzyżek, 2024). Similarly, inhibition of *H. pylori* efflux activity by bifidobacterial metabolites represents a biologically plausible mechanism. However, direct molecular evidence demonstrating that *Bifidobacterium* specifically inhibits HefA or other *H. pylori* efflux systems remains insufficient (Gong et al., 2024). These proposed effects should therefore be regarded as hypotheses requiring validation through measurements of efflux-pump expression, intracellular antibiotic accumulation, biofilm biomass and viability, and antimicrobial susceptibility after exposure to defined bifidobacterial strains or their metabolites (Figure 2).

### 5.5 Direct intragastric competition and indirect gut-mediated effects

Direct intragastric competition requires probiotic bacteria to survive gastric acidity, remain in close proximity to the gastric mucosa, and compete with *H. pylori* for local adhesion sites or nutrients. Because many *Bifidobacterium* strains are primarily adapted to the intestinal environment, persistent high-abundance colonization of the stomach may be limited. Therefore, direct competition for gastric mucosal binding sites is likely to be strain-dependent and should not be considered a universal mechanism of bifidobacterial action. Nevertheless, experimental observations suggest that selected preparations may influence *H. pylori* adhesion. For example, *B. animalis* BD7 combined with fucoidan inhibited *H. pylori* adhesion and reduced urease activity (Guo et al., 2026), although the effects of this combined intervention cannot be attributed solely to the bifidobacterial strain. In contrast, indirect gut-mediated effects do not require persistent colonization of the gastric mucosa. Bifidobacteria may alter the distal intestinal microbial environment through production of microbial metabolites, modulation of host immune responses, reinforcement of epithelial barrier function, and restoration of microbiota composition after antibiotic treatment (Yuan et al., 2025; Jiang et al., 2025; Ronkainen et al., 2025). Changes in the abundance of bifidobacteria and other intestinal taxa have also been observed during and after *H. pylori* eradication therapy. These distal intestinal effects may influence the broader gastrointestinal environment and host response and may therefore contribute indirectly to *H. pylori* treatment outcomes.

Accordingly, direct gastric competition and indirect regulation through the distal gut microbiota should be regarded as complementary but biologically distinct mechanisms. For many *Bifidobacterium* strains, indirect gut-mediated regulation may be more plausible than persistent direct competition within the gastric niche. This distinction also provides a rationale for investigating postbiotics and other microbial-derived products that could retain biological activity without requiring viable bifidobacteria to colonize the stomach (Figure 2).

## 6 Discussion

The central argument of this review is that *Bifidobacterium* can serve as a meaningful adjunctive strategy to counteract antibiotic-resistant *H. pylori*. While the evidence summarized above supports this position, a deeper examination reveals several conceptual and practical challenges that complicate its straightforward translation into clinical practice. These challenges involve not only the antibacterial activity of *Bifidobacterium*, but also its survival during treatment, its interaction with the gastrointestinal environment, and differences between individual patients.

A fundamental tension lies at the heart of the probiotic-antibiotic combination: antibiotics are designed to kill or inhibit bacteria, yet we are asking live *Bifidobacterium* to survive, colonize, and exert effects in a patient simultaneously receiving high-dose bactericidal agents and bismuth. Clinical studies have used probiotics before, during, or after eradication therapy, and the optimal timing of supplementation remains uncertain (Shin et al., 2024; Jiang et al., 2023; Zhang Y. et al., 2025). One resolution to this paradox is the concept of postbiotics—non-viable bacterial preparations or their metabolites that retain biological activity without requiring live cells to survive antibiotic exposure (Ivashkin et al., 2024; Fauzia and Tuan, 2024). If the key anti-*H. pylori* effects of *Bifidobacterium* are mediated by secreted organic acids, antimicrobial peptides, or immunomodulatory surface molecules, then heat-killed bacteria or purified metabolites could theoretically achieve the same benefit without the viability constraint. This shifts the research agenda from “which *Bifidobacterium* strain?” to “which *Bifidobacterium*-derived product?” However, bacterial viability is only one part of the problem. The site and mode of action of *Bifidobacterium* also need to be considered.

The competitive exclusion model that *Bifidobacterium* physically occupies niches or depletes nutrients needed by *H. pylori*, is appealing but likely oversimplified. *H. pylori* colonizes the gastric mucosa, whereas most *Bifidobacterium* species are primarily adapted to the intestinal environment. In addition, the strongly acidic gastric environment limits the survival and persistent colonization of many probiotic strains, making sustained direct competition for the same gastric mucosal niche less likely (He et al., 2023; Koga, 2022). A more plausible ecological mechanism is indirect modulation: *Bifidobacterium* alters the broader gastrointestinal environment through SCFA production, immune signaling, or mucus composition in ways that render the stomach less permissive to *H. pylori* colonization (Bordin et al., 2022; Li et al., 2024; Ivashkin et al., 2024). This distinction matters for therapeutic design. If *Bifidobacterium* acts primarily via distal gut-derived signals rather than local gastric occupancy, then gastric acid viability becomes less critical, delivery methods can prioritize gut delivery over gastric survival, and prebiotics that boost endogenous *Bifidobacterium* in the gut may be as effective as exogenous probiotic supplementation (Bedu-Ferrari et al., 2022). This gut–stomach interaction also suggests that pre-existing intestinal and metabolic disorders may influence the response to *H. pylori* eradication therapy.

Co-morbid intestinal and metabolic diseases may complicate *H. pylori* treatment. Many patients already have gastrointestinal symptoms or changes in the gut microbiota before eradication therapy begins. Irritable bowel syndrome is one example. Several studies have examined the relationship between *H. pylori* infection and irritable bowel syndrome, although the findings remain inconsistent. One meta-analysis of 31 studies involving 21,867 participants reported a positive association (Wang C. et al., 2021). At the same time, antibiotic-based eradication therapy can cause short-term changes in the intestinal microbiota. A systematic review and meta-analysis found that intestinal microbial diversity decreased early after *H. pylori* treatment in most included studies (Du L. et al., 2024). These changes may be more important in patients who already have intestinal symptoms or microbiota imbalance. Endocrine and metabolic diseases can also affect treatment. Type 2 diabetes mellitus is an important example. A meta-analysis found that patients with type 2 diabetes had a higher risk of *H. pylori* eradication failure than patients without diabetes (OR 2.59, 95% CI 1.82–3.70) (Song et al., 2021). Recent clinical evidence also suggests that metabolic control may influence treatment response. In patients with type 2 diabetes, higher HbA1c levels have been associated with an increased risk of eradication failure (Zhang J. et al., 2025). Glycemic control, previous antibiotic exposure, treatment adherence, and changes in gastrointestinal function may contribute to this difference. These co-morbidities may therefore increase treatment difficulty and also create a stronger need for supportive strategies that can protect the gastrointestinal environment during eradication therapy.

Probiotics may help address some of these treatment problems. First, probiotics may reduce antibiotic-related intestinal dysbiosis. A meta-analysis showed that some bacterial changes after *H. pylori* therapy were less marked in patients who received probiotics (Du L. et al., 2024). Second, probiotics may reduce gastrointestinal adverse effects and improve treatment adherence. A 2024 umbrella review of 28 meta-analyses based on 534 randomized trials found that probiotic supplementation was associated with a higher eradication rate (RR 1.10, 95% CI 1.06–1.14) and a lower risk of overall adverse effects (RR 0.54, 95% CI 0.42–0.70) (Yang et al., 2024). These effects may be useful for patients who already have intestinal symptoms or metabolic co-morbidities. Third, some *Bifidobacterium* strains may directly protect the gastric mucosa. In a recent experimental study, *B. animalis* subsp. lactis BB18 reduced *H. pylori* load, gastric mucosal damage, and inflammatory cytokine levels in infected Mongolian gerbils (Zheng et al., 2024). These findings suggest that *Bifidobacterium* may reduce mucosal inflammation and support epithelial barrier function. These effects could help limit the mucosal damage that contributes to *H. pylori*-associated ulcer formation. However, direct clinical evidence that probiotics can prevent gastric ulcers in humans is still limited. Future trials should therefore include ulcer healing, ulcer recurrence, and mucosal injury as clinical outcomes. Even if probiotics improve the intestinal environment and gastric mucosal protection, one major microbiological problem may still limit complete eradication: the dormant coccoid form of *H. pylori*.

Perhaps the most significant gap in the current literature is the question of whether *Bifidobacterium* can affect the dormant coccoid form of *H. pylori*. Under antibiotic pressure or environmental stress, *H. pylori* readily transitions from its metabolically active spiral form to a viable-but-non-culturable coccoid state (Sadeghloo et al., 2025; Ramezani et al., 2025). These dormant cells have markedly reduced metabolic activity, rendering some antibiotics less effective, are often located deep within gastric crypts physically shielded from treatment, and can revert to active spiral forms upon withdrawal of antibiotic pressure, driving relapse (Sadeghloo et al., 2025). Virtually all existing studies on *Bifidobacterium*-*H. pylori* interactions have been conducted on actively growing, spiral-form *H. pylori in vitro* or in animal models. Whether *Bifidobacterium* or its metabolites can prevent the spiral-to-coccoid transition, eradicate already-formed coccoid forms, or block their reactivation remains entirely unknown. Probiotic supplementation may improve eradication rates, but its effect on long-term relapse remains uncertain (Tanashat et al., 2025; Yang et al., 2024; Zhang et al., 2020). If coccoid forms are the reservoir of persistent infection, and if *Bifidobacterium* does not affect them, then the current strategy is fundamentally incomplete. This limitation also shows why the same probiotic strategy may not work equally well in every patient.

Looking forward, the field must move away from “one-size-fits-all” probiotic supplementation toward precision microbiota engineering. The composition of the gut microbiota, the presence of specific *H. pylori* resistance mutations, host immune status, and dietary factors all vary substantially across individuals, yet current clinical trials treat probiotics as a uniform add-on with little stratification (Brumfield et al., 2020; Wang et al., 2022; Zhao et al., 2025). Future approaches might include baseline microbiota profiling to identify patients with pre-existing low *Bifidobacterium* abundance who might benefit most, strain selection based on local *H. pylori* resistance patterns, synbiotic combinations pairing *Bifidobacterium* with prebiotics such as GOS or inulin that selectively enhance its activity (Bedu-Ferrari et al., 2022), and multi-omics monitoring during therapy to dynamically track microbiota responses (Wang et al., 2022). However, precision treatment still faces several practical barriers before these approaches can be used routinely.

Personalized treatment of *H. pylori* is promising, but several problems still limit its routine use. Antibiotic susceptibility testing is not available in all hospitals. Culture-based testing requires endoscopy and suitable biopsy samples, and *H. pylori* culture can be difficult. Molecular tests are faster, but many tests detect only a limited number of known resistance mutations. They may also miss uncommon resistance mechanisms or bacterial heteroresistance. Host factors create another source of variation. For example, *CYP2C19* genotype can affect the metabolism of some PPIs and can influence eradication success. A 2022 meta-analysis showed that eradication rates differed between *CYP2C19* metabolizer groups, especially when omeprazole- or lansoprazole-based regimens were used (Zhao et al., 2022). Previous antibiotic exposure, drug allergy, treatment history, adherence, diet, co-morbidities, and baseline microbiota can further change treatment response. For this reason, personalized treatment may need to combine bacterial, host, and microbiota information rather than rely on a single resistance test.

Omics technologies may provide a way to integrate these different factors. Pathogen genomics and next-generation sequencing can identify multiple resistance-associated variants in a single assay, while host pharmacogenomics can identify variants such as *CYP2C19* that influence the efficacy of acid-suppressive therapy (Zhao et al., 2022; Min et al., 2024). Metagenomic analysis can describe the gut microbiota and the antibiotic resistome before and after treatment. Metabolomics can identify changes in microbial and host metabolites that may be linked to treatment response (Zhao et al., 2025; Suo et al., 2025; Wenhui et al., 2022). These data could eventually be combined to select antibiotics, acid suppressants, and probiotic strains for individual patients. A 2024 study developed a next-generation sequencing panel that assessed *H. pylori* resistance-related variants together with genes involved in PPI metabolism and then used these data to suggest individualized eradication regimens (Min et al., 2024). This study included only a small number of patients, so larger validation studies are still needed. Recent metagenomic data also show that eradication therapy can change the intestinal resistome and increase mobile antibiotic resistance genes (Zhao et al., 2025). Future studies should therefore evaluate both eradication success and the broader effects of treatment on the host and gastrointestinal microbiota.

## 7 Conclusion

Antibiotic resistance in *H. pylori* has become a major obstacle to successful eradication, creating an urgent need for adjunctive strategies. Current evidence suggests that *Bifidobacterium* may support eradication through direct growth inhibition, modulation of mucosal inflammation, protection of epithelial barrier function, and regulation of the gastrointestinal microbial environment. Clinical studies and meta-analyses also indicate that probiotic supplementation can reduce treatment-related adverse effects and may improve eradication rates, although the magnitude of benefit varies according to strain, regimen, timing, and study population.

Several proposed mechanisms require further validation. In particular, direct inhibition of *H. pylori* efflux pumps or AI-2 signaling by bifidobacterial metabolites has not been conclusively demonstrated, and direct clinical evidence in patients infected with confirmed antibiotic-resistant strains remains limited. Future studies should prioritize strain-specific trials, direct molecular testing of proposed biofilm and efflux mechanisms, and assessment of postbiotic preparations. Particular attention should be given to determining whether *Bifidobacterium* or its postbiotics can inhibit the spiral-to-coccoid transition, eliminate dormant coccoid cells, or prevent their reactivation after antibiotic treatment.
