# Correction: Potential mechanisms of *Bifidobacterium*-mediated synergistic antibiotic eradication of drug-resistant *Helicobacter pylori*

**DOI:** 10.3389/fmicb.2026.2031673

**Published:** 2026-10-29

**Authors:** Linna Wu, Xin Chen, Mingyu Cui, Shuang Yan, Lijuan Wei, Ruifeng Duan

**Affiliations:** Department of Gastroenterology and Digestive Endoscopy Center, The Second Hospital of Jilin University, Changchun, China

**Keywords:** antibiotic resistance, bifidobacteria, *Helicobacter pylori*, probiotic, synbiotics

Author Xin Chen was erroneously omitted as equal contributing author.

The original version of this article has been updated.

